# One-year Success Rate of Selective Laser Trabeculoplasty in Pseudoexfoliative Glaucoma: A Prospective Study

**DOI:** 10.18502/jovr.v21.17046

**Published:** 2026-02-19

**Authors:** Behzad Fallahi Motlagh, Amin Arasteh, Ali Mostafaie, Hakimallah Ghalamara, Farhad Najafzadeh

**Affiliations:** Department of Ophthalmology, Nikookari Eye Hospital, Tabriz University of Medical Sciences, Tabriz, East Azerbaijan, Iran

**Keywords:** PEXG, Pseudoexfoliative Glaucoma, Selective Laser Trabeculoplasty, SLT

## Abstract

**Purpose:**

To evaluate the 1-year effectiveness of selective laser trabeculoplasty (SLT) in patients with pseudoexfoliative glaucoma (PEXG).

**Methods:**

This prospective, single-arm, non-randomized interventional study involved 40 eyes from 40 patients with PEXG with no history of glaucoma surgery and laser trabeculoplasty. All participants underwent a single session of 360º SLT. Follow-up visits were conducted at 2 hours, 1 day, 1 week, and at 1, 2, 3, 4, 5, 6, and 12 months after the procedure. The intraocular pressure (IOP), the number of anti-glaucoma medications (NOM), and the 1-year success rate were investigated as primary outcome measures; while the incidence of adverse effects was considered the secondary outcome. Treatment success was defined as a ≥ 20% decrease in IOP compared to baseline.

**Results:**

The baseline IOP was 22.19 ± 2.08 mmHg, and the mean NOM was 3.12 ± 0.55 before the SLT procedure. The mean IOP was significantly lower at all follow-up visits than at baseline, whereas the mean NOM remained unchanged throughout the follow-up period. The mean IOP values at 1, 3, 6, and 12 months after SLT were 18.02, 16.92, 16.47, and 15.31 mmHg, respectively. The overall 1-year success rate was 85%, with an average IOP reduction of 31.0% relative to baseline. The most common adverse event following SLT was mild anterior chamber inflammation, which occurred in 32.5% of eyes.

**Conclusion:**

The SLT procedure could significantly reduce IOP in patients with PEXG with minimal adverse effects. The 1-year outcomes were favorable, suggesting that SLT may serve as an effective primary or complementary treatment for PEXG.

##  INTRODUCTION

Glaucoma is the primary cause of permanent blindness globally, affecting approximately 60 million individuals.^[1]^ Open-angle glaucoma (OAG) is the most common form, with primary open-angle glaucoma (POAG) accounting for about 75% of cases.^[2]^ Among the secondary OAG subtypes, pseudoexfoliative glaucoma (PEXG) is the most prevalent. The prevalence and incidence of PEXG vary across ethnicities and populations. PEXG differs from POAG due to its poorer long-term prognosis, mainly attributable to reduced responsiveness to anti-glaucoma medications.^[3]^ A population-based study of people aged 40-80 in central Iran revealed a 4.4% prevalence of glaucoma, with PEXG accounting for 0.4%; the presence of pseudoexfoliation was the leading risk factor for PEXG incidence.^[4]^ Early diagnosis and intensive management may improve outcomes in these patients.

**Figure 1:**
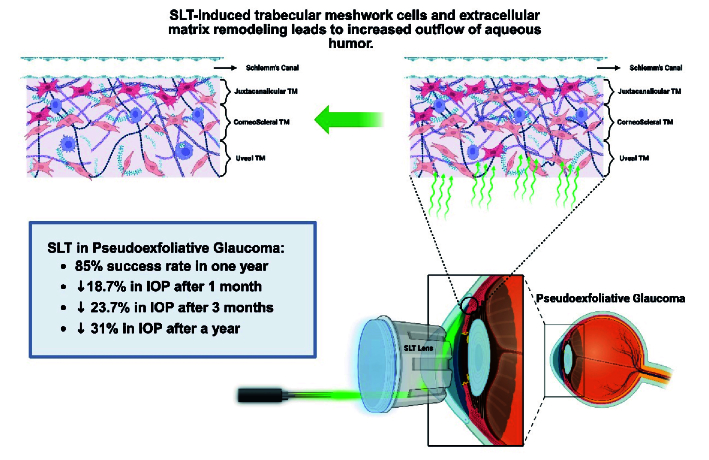
A schematic mechanism of selective laser trabeculoplasty in glaucoma.

Therapeutic options for all glaucoma subtypes range from medical treatments to invasive surgical interventions. Laser trabeculoplasty represents a less invasive procedure that reduces intraocular pressure (IOP) by increasing aqueous outflow through trabecular meshwork (TM) remodeling.

For the first time, selective laser trabeculoplasty (SLT) principles were introduced in 1983 to selectively target pigmented TM structures. The laser energy is thought to induce remodeling of the TM, thereby reducing resistance to aqueous humor outflow [Figure 1].^[5]^. This remodeling involves various mechanisms, including modulation of extracellular matrix protein gene expression, enhanced cytokine release, induction of apoptosis and necrosis, and stimulation of monocyte migration and activation.^[6]^

SLT has been used as an effective therapeutic option for various glaucoma subtypes, including normal-tension glaucoma, POAG, PEXG, pigmentary glaucoma, and steroid-induced glaucoma.^[7]^ Reported IOP reductions in POAG range from 6.9% to 35.9%, depending on disease severity.^[8]^ While SLT may provide long-term IOP control as a primary or additive treatment, its success may vary across OAG subtypes.^[9]^

This study aims to evaluate the 1-year success rate and IOP-lowering effect of SLT—as an adjunct treatment—in patients with PEXG.

##  METHODS

### Patients

This prospective single-arm, nonrandomized interventional study was conducted on patients with PEXG at Nikookari Eye Hospital in Tabriz, Iran, between 2015 and 2017. The study aimed to investigate the therapeutic effect of SLT on this population over a year.

We enrolled a total of 40 patients diagnosed with PEXG who met the inclusion and exclusion criteria and provided written informed consent. Pseudoexfoliation (PEX) syndrome was defined as the presence of pseudoexfoliative material on the pupillary margin or the anterior surface of the crystalline lens. PEXG was defined as structural and/or functional evidence of glaucomatous optic neuropathy, including an increased cup-to-disk (C/D) ratio, retinal nerve fiber layer (RNFL) atrophy, or visual field defects in a patient with clinically confirmed PEX syndrome.

The inclusion criteria were detailed as follows: Patients with PEXG who failed to achieve target IOP—defined as IOP > 21 mmHg or documented glaucoma progression despite IOP ≤ 21 mmHg—under maximal tolerated topical therapy, or those who required discontinuation of some medications due to side effects, were included in the study. Patients were excluded if they had any of the following: Angle closure component on gonioscopy, history of ocular trauma, previous argon laser trabeculoplasty (ALT) or SLT, history of past ocular surgery except uncomplicated cataract surgery performed more than 6 months prior, presence of other ocular conditions such as diabetic retinopathy, significant cataract, uveitis, and corneal dystrophies, and unwillingness to participate further in the study.

Only one eye per patient was included in the study. If both eyes met the inclusion criteria, the eye with the higher IOP was selected. The fellow eye was excluded, even though it later met the criteria during follow-up. Patients who did not complete the 12-month follow-up after SLT were excluded from the study and the final analysis.

### Intervention

All patients received a single 360º session of selective SLT, consisting of approximately 100 laser applications with a spot size of 400 µm, duration of 3 nanoseconds, and initial energy of 0.6 mJ.

The energy was titrated in 0.1 mJ increments according to TM pigmentation and tissue response, up to the maximum energy level that did not induce bubble formation (cavitation). All procedures were performed by the same ophthalmologist using a Q-switched Nd: YAG laser system (Ellex Solo, Ellex Medical Pty. Ltd., Adelaide, Australia). This was conducted under topical anesthesia with two drops of tetracaine hydrochloride 0.5% ophthalmic solution, instilled 5 minutes before the procedure, and a Latina goniolens (Ocular Instruments Inc., WA, USA).

No routine anti-inflammatory medication was prescribed for patients after the SLT procedure. However, in cases with significant pain or inflammation, a drop of loteprednol 0.5% ophthalmic solution was administered every 8 hours for 1 week. Patients were instructed to continue their pre-existing anti-glaucoma medications after SLT unless otherwise indicated.

**Table 1:** Number of anti-glaucoma medications (NOM) and intraocular pressure (IOP) at baseline and follow-up visits compared to baseline

**Time point**	**NOM (** ***n*** **) (mean) ± SD**	**Inter-visit comparison (** ***P*** **-value)**	**IOP (mmHg) (mean) ± SD**	**Comparison to baseline (** ***P*** **-value)**
Baseline	(3.12) ± 0.55	0.413	(22.19) ± 2.08	*–*
2 hours	(3.02) ± 0.60		(21.90) ± 2.29	< 0.0001
1 day	(3.02) ± 0.60		(18.74) ± 2.27	< 0.0001
1 week	(3.02) ± 0.60		(18.98) ± 2.47	< 0.0001
1 month	(3.02) ± 0.60		(18.02) ± 5.31	< 0.0001
2 months	(2.95) ± 0.56		(17.90) ± 3.60	< 0.0001
3 months	(2.89) ± 0.57		(16.92) ± 2.91	< 0.0001
4 months	(2.86) ± 0.55		(17.38) ± 2.19	< 0.0001
5 months	(2.86) ± 0.55		(16.97) ± 2.18	< 0.0001
6 months	(2.86) ± 0.55		(16.47) ± 1.97	< 0.0001
1 year	(2.86) ± 0.55		(15.31) ± 1.84	< 0.0001

**Figure 2:**
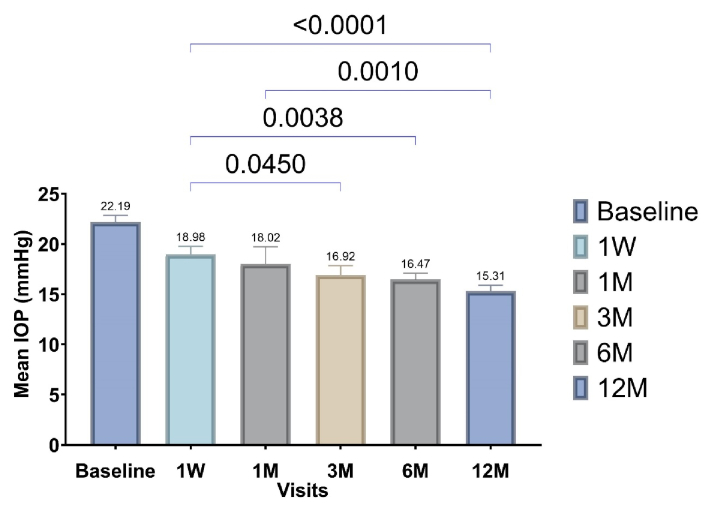
Pairwise comparisons of the mean IOP between baseline and different follow-up visits. The *P*-values of pairwise comparisons are shown above the blue brackets.

**Figure 3:**
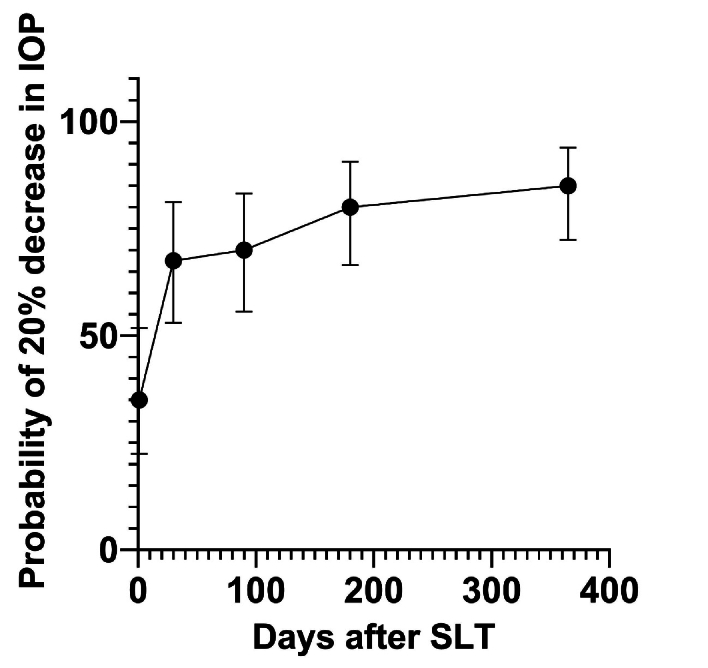
Kaplan–Meier curve showing the probability of achieving at least a 20% reduction in IOP compared with baseline over the follow-up period. The bars show 95% confidence intervals.

### Visits and Target Outcomes

Baseline IOP and the NOM were recorded before SLT. The follow-up visits were scheduled at 2 hours, 1 day, 1 week, 1 month, 2 months, 3 months, 4 months, 5 months, 6 months, and 12 months after the procedure. At each visit, IOP, NOM, and any adverse events were evaluated. Additionally, all patients underwent a thorough slit-lamp examination, fundoscopy, and uncorrected visual acuity (UCVA) assessment at baseline and at follow-up visits. When topical anesthesia was achieved with tetracaine eye drops, IOP was measured using a Goldmann applanation tonometer by the same examiner between 8 am and 12 pm at the glaucoma clinic.

The primary outcome was the change due to SLT in IOP and NOM at each follow-up visit compared to baseline, as well as the 1-year success rate, defined as ≥ 20% reduction in IOP relative to baseline. The secondary outcome was to evaluate the post-SLT adverse effects among patients with PEXG.

### Statistical Analysis

All statistical analyses were conducted using SPSS version 22.0 (IBM Corp., Armonk, NY, USA). The normality of data distribution was assessed using the Shapiro–Wilk test. Repeated-measures ANOVA was used to compare the variables of the follow-up visits with baseline measurements, followed by Tukey's post hoc test for pairwise comparisons. A *P*-value < 0.05 was considered statistically significant. All figures are created using GraphPad Prism version 10.0 for Windows (GraphPad Software, San Diego, CA, USA), and the graphical abstract was prepared using BioRender.com.

### Ethical Considerations

This study has been conducted in accordance with the ethical guidelines of the Declaration of Helsinki. The study protocol was approved by the Research Ethics Committee of Tabriz University of Medical Sciences (Approval Code: 93135). All participants provided written informed consent prior to enrollment.

##  RESULTS

A total of 40 eyes from 40 patients (21 men [52.5%] and 19 women [47.5%]) with a mean age of 64.36 ± 5.76 years were included in this study. The baseline IOP in the target population was 22.19 ± 2.08 mmHg, and the mean NOM was 3.12 ± 0.55 before SLT.

There was no significant change in NOM at any follow-up visit after SLT (*P *= 0.413). In contrast, IOP showed a statistically significant decrease at all follow-up visits compared with baseline (*P* < 0.0001). Based on the post hoc analysis, the only follow-up time point without a significant difference from baseline was 2 hours after SLT (*P >* 0.999). The mean IOP reduction at 1 week, 1 month, 3 months, 6 months, and 12 months after SLT compared to baseline was 3.21 mmHg (95% CI, 1.17-5.24), 4.17 mmHg (95% CI, 2.13-6.20), 5.27 mmHg (95% CI, 3.23-7.30), 5.72 mmHg (95% CI, 3.68-7.75), and 6.88 mmHg (95% CI, 4.84-8.91), respectively. Relative to baseline, IOP decreased by 18.7% (95% CI, 9.5-27.9), 25.7% (95% CI, 16.5-34.9), and 31.0% (95% CI, 21.8-40.1) at month 1, month 6, and month 12, respectively. Table 1 presents details on NOM and IOP data across all follow-up visits.

Pairwise comparisons among the 1-week, 1-month, 3-month, 6-month, and 12-month follow-ups after SLT revealed significant differences in four comparisons.

The mean IOP at 12 months was significantly lower than at 1 week and 1 month (mean difference: 3.67 mmHg [95% CI, 1.63-5.70] and 2.71 mmHg [95% CI, 0.67-4.74], respectively). Additionally, IOP decreased significantly at 3 months and 6 months compared with 1 week (mean difference: 2.06 mmHg [95% CI, 0.02-4.09] and 2.51 mmHg [95% CI, 0.47-4.54], respectively).

Figure 2 illustrates these pairwise comparisons in more detail.

After a 12-month follow-up, 85% (*n* = 34) of patients achieved at least a 20% reduction in IOP from baseline. The remaining six patients who did not meet the success criterion underwent trabeculectomy due to poor compliance with topical medications, although no sign of glaucoma progression was observed. The Kaplan–Meier survival curve [Figure 3] demonstrates the probability of achieving at least a 20% IOP reduction during the follow-up period.

By the first postoperative day, 35% (95% CI, 22.4-51.8) of eyes had achieved this reduction, increasing to 67.5% (95% CI, 53.0-71.2) by 1 month, with an additional 17.5% achieving success thereafter.

The most common adverse event after SLT was mild anterior chamber inflammation (defined as ≤ 1+ cells or flare according to the Standardization of Uveitis Nomenclature [SUN] grading). It occurred in 13 eyes (32.5%) and responded well to topical loteprednol etabonate 0.5% eye drops every 8 hours for 1 week. Ten eyes (25%) experienced a transient IOP elevation, lasting < 1 hour and not exceeding 5 mmHg above baseline. Additionally, eight patients (20%) reported transient ocular pain or discomfort lasting < 15 minutes after SLT. No major or sight-threatening complications were observed during the study.

At the end of the 1-year follow-up, although six patients who did not achieve a 20% IOP reduction showed no signs of glaucoma progression, they underwent trabeculectomy due to poor compliance with topical medications.

##  DISCUSSION

Our study demonstrated that patients with PEXG achieved good therapeutic outcomes after SLT. Eighty-five percent of cases experienced at least a 20% reduction in IOP after a year, with a mean IOP reduction of 31%. However, the NOM did not change from baseline in follow-up visits. Most patients (67.5%) reached a 20% reduction in IOP in the first month after SLT. The most common adverse event observed was mild anterior chamber inflammation (32.5%).

PEX syndrome affects 10-15% of the general population over 60 years of age, and PEXG accounts for approximately one-quarter of OAG cases. The pathophysiology of PEXG involves the accumulation of pseudoexfoliative material within the TM and uveoscleral pathways, leading to impaired aqueous outflow. Structural changes in the lamina cribrosa may further increase vulnerability to the elevated IOP.^[10]^ Compared to POAG, PEXG typically occurs at older ages, has a poorer prognosis, and shows more rapid progression. In addition to the predominantly open-angle pathology, a mixed mechanism involving angle closure may occur in PEXG due to the zonular weakness. Beyond medical therapy, surgical interventions such as trabeculectomy, drainage tubes, or minimally invasive glaucoma surgeries can mitigate disease progression. However, as a less invasive procedure, SLT plays a significant therapeutic role in patients with PEXG before a surgical procedure.^[11]^

The literature review shows that SLT can serve as both a primary and adjunctive therapeutic option for exfoliative glaucoma.^[12]^ The LiGHT trial suggested that SLT could be considered a primary treatment for ocular hypertension and OAG, rather than merely a complement to conventional medical therapy, with a long-term success rate of 70%.^[13]^ A systematic review comparing SLT with medical therapy reported no significant differences in IOP reduction, but it found that patients treated with SLT required fewer medications.^[14]^

Both ALT and SLT have been shown to provide comparable long-term IOP-lowering effects in patients with PEXG.^[15,16]^ A prospective study of 57 eyes with PEXG reported a mean IOP reduction of 8.2 mmHg and a decrease of 0.5 medications 1 year after SLT.^[17]^ Our results were similar regarding IOP reduction (6.9 mmHg), although the number of medications did not change post-SLT. The 1-year success rate in our study was 85% compared to 58.1% in a retrospective Korean study,^[18]^ and 50% in another Korean study without a significant change in medications.^[19]^A recent retrospective study in the United States reported 1-year success rates of 56.9% for POAG and 49.3% for pigmentary glaucoma, with baseline IOP being the main predictor of reduction at 12 months.^[20]^ Variations in success rates may be influenced by factors such as ethnicity, TM pigmentation, and SLT extent (180º vs. 360º), although some studies do not support this hypothesis.^[21,22]^

Regarding predictors of success, baseline IOP < 15 mmHg may reduce SLT efficacy, particularly when used as a primary treatment.^[23]^ Male sex, higher baseline IOP, prior medical therapy, and successful SLT in the fellow eye have also been associated with higher success rates.^[24]^ Failure in PEXG may be related to insufficient modulation of metalloproteinase inhibitors relative to metalloproteinases in the TM.^[25]^

Some studies suggest that the IOP-lowering effect of SLT in PEXG is maintained for up to 21 months, compared to 30 months in POAG.^[26]^ A comparative study reported six-month success rates of 94.1% in patients with PEXG and 75% in those with POAG, which decreased substantially after 1 year to 25.0% and 29.1%, respectively.^[27]^ In our study, success rates stabilized after the third month of follow-up, with some patients showing a gradual decrease in IOP over time (15.31 mmHg at 12 months vs. 18.02 mmHg at 1 month). Although the reduction gradient of IOP became less pronounced with extended follow-up, it did not cease until the 1-year follow-up period. This continued reduction may reflect dynamic TM remodeling induced by SLT and could be influenced by disease stage.

Weinand and Althen reported a 27.8% decrease in IOP 2 years after SLT; however, the corresponding value at the 1-year follow-up was 24.3%.^[28]^ Although the IOP changes after the third month in our study may not be clinically significant (mean difference: 1.61 mmHg), they were statistically significant. Variations in the timing of IOP measurements and the interval between taking the anti-glaucoma medication and IOP assessment should be considered as potential confounding factors. Diurnal fluctuations in IOP are well recognized in the general population, with peak levels occurring between 6 am and 10 am.^[29]^ Moreover, some physiological changes, such as seasonal variation, may influence IOP; patients tend to experience higher IOP values in winter than in summer.^[30]^ Therefore, the changes observed after the third month of follow-up in our study should not be overemphasized in clinical interpretation.

Some studies have reported a more substantial IOP-lowering effect in PEXG compared to POAG after 1 year (6.1 vs. 4.4 mmHg decrease).^[31]^ Meanwhile, a prospective study on OAG in Iran showed no significant difference between the two study groups 16 months after SLT (16.7% vs. 16.6%). In that study, NOM also did not change after SLT, and the overall success rate at 16 months was 56.6%.^[32]^ Although our findings regarding the effect of SLT on NOM were similar, our study demonstrated substantially higher 1-year success rates and IOP reductions than that other study (85% and 31%, respectively). It should be noted that our study included a larger number of PEXG cases compared to that other study (40 vs. 22), which may partially explain this difference.

Regarding the temporary effect of SLT, one study revealed a higher efficacy for annual low-power SLT in controlling ocular hypertension compared with conventional SLT.^[33]^ Similarly, another study on PEXG reported a significant reduction in IOP without notable complications after the second and third SLT sessions.^[34]^ It has also been suggested that SLT could be effective for patients with PEXG following ALT failure, with a 9-month success rate of 42%.^[35]^ Nevertheless, potential postoperative side effects should be carefully monitored when repeating the SLT procedure.

In our study, the most common complications observed in patients after SLT were mild anterior chamber inflammation (32.5%), a transient IOP rise of less than 5 mmHg (25%), and transient ocular pain and discomfort (20%). A comparable study reported post-SLT inflammation in 40.9% and pain in 18.2% of PEXG cases, noting that IOP spikes < 5 mmHg were not considered adverse events.^[32]^ The LiGHT trial identified discomfort as the most common adverse event (23.1%), followed by altered vision (5.9%), photophobia (5.6%), IOP spike (1.7%), refractive change (1.1%), hyperemia (0.8%), and inflammation (0.3%).^[36]^ However, another study reported higher incidences of mild anterior uveitis (71.4%), discomfort and pain (65.7%), and IOP spikes (14.3%).^[37]^ Although rare, serious complications such as uncontrolled IOP requiring glaucoma drainage implant or corneal decompensation necessitating corneal transplantation have been reported in case series.^[38]^ Overall, SLT is generally safe, with most complications transient and easily treatable. However, during the post-SLT examination, it is essential to differentiate true anterior chamber inflammation from floating pigment granules, since each requires a distinct management approach. Variability in the reported incidence of anterior uveitis may be attributable to differences in laser energy parameters and the heterogeneity of glaucoma subtypes.

The non-randomized, single-arm design of our study limits the statistical power of the findings, although the relatively long-term follow-up period partially mitigates this limitation. Additional limitations include the absence of detailed SLT energy parameters and the lack of more functional and structural assessments (e.g., perimetry and optical coherence tomography) during the follow-up visits. Further randomized clinical trials with larger sample sizes are warranted to provide more robust evidence on the therapeutic efficacy and long-term outcomes of SLT in patients with PEXG.

In summary, based on our findings, 85% of patients achieved at least a 20% reduction in IOP after 1 year, with a mean decrease of approximately 31%. Notably, no significant vision-threatening side effects were observed. This significant success rate and substantial reduction in IOP suggest that SLT is a promising therapeutic option for PEXG, even as a primary treatment. Moreover, the delayed IOP reduction observed in some patients may reflect structural changes and tissue remodeling within the TM, warranting further investigation in future studies.

##  Financial Support and Sponsorship

None.

##  Conflicts of Interest

None.
